# Relationship between heart rate variability and echocardiography indices of cardiac function in healthy individuals

**DOI:** 10.1111/cpf.12910

**Published:** 2024-11-05

**Authors:** Mushidur Rahman, Sophie L. Russell, Nduka C. Okwose, Charles J. Steward, Helen Maddock, Prithwish Banerjee, Djordje G. Jakovljevic

**Affiliations:** ^1^ Research Centre for Health and Life Sciences, Institute for Health and Wellbeing, Coventry University Coventry UK; ^2^ Department of Cardiology University Hospitals Coventry and Warwickshire NHS Trust Coventry UK

**Keywords:** echocardiography, heart rate variability, speckle tracking echocardiography

## Abstract

**Purpose:**

This study evaluated the relationship between HRV and echocardiography indices of cardiac function.

**Methods:**

Healthy individuals (*N* = 30) aged 33 ± 10 years old, underwent short‐term resting HRV assessment and transthoracic echocardiography with speckle tracking analysis. Time domain ‐ (i.e. R‐R interval, root mean square of successive RR interval difference (RMSSD), standard deviation of normal RR intervals (SDNN) and frequency domain‐measures of HRV (i.e. high‐frequency power (HF), low‐frequency power (LF), high‐frequency normalised (HFnorm) and low‐frequency normalised (LFnorm)). Echocardiography indices of cardiac function included; Left ventricular ejection fraction (LVEF), left‐ and right‐ventricular global longitudinal strain (LV‐GLS, and RV GLS), left atrial strain: left atrial reservoir (LA_res_), left atrial conduit (LA_con_) and left atrial contraction (LA_CT_).

**Results:**

The mean values for HRV time‐domain measures were: R‐R (991 ± 176 ms), SDNN (50.9 ± 21.5 ms), and RMSSD (46.8 ± 29.4 ms); and frequency‐domain: LF (727 ± 606 ms^2^), HF (415 ± 35 ms^2^), LFnorm (56 ± 19.4) and HFnorm (36.5 ± 18.8). Mean values for indices of cardiac function were LVEF (59.9% ± 2.8%), LV‐GLS (19.2% ± 1.4%), RV‐GLS (21.7% ± 2.7%), LA_res_ (36.8% ± 6.99%), LA_con_ (26.2% ± 6.95%) and LA_CT_ (12.3% ± 3.56%). There was a significant negative relationship between HF and LV‐GLS (*r* = −0.47, *p* = 0.01) and RMSSD and LVEF (*r* = −0.39, *p* = 0.03) respectively.

**Conclusion:**

Heart rate variability measures such as high frequency power and RMSSD are associated with left ventricle systolic function in healthy individuals.

## 1 INTRODUCTION

Heart rate variability (HRV) is the variation in time between consecutive heart beats (Shaffer and Ginsberg, 2017). HRV is an indicator of autonomic nervous system function and reflects the interplay between sympathetic and parasympathetic nervous systems (Evans et al., 2013). HRV has gained a considerable amount of interest for its utility as a simple noninvasive measure to better understand cardiovascular physiology in health and disease (Thayer et al., 2010). It was also reported to be a strong predictor of mortality in the general and clinical population (Jarczok et al., 2022). It may play an important role in cardiovascular disease risk stratification and prognosis (Billman, 2011; Jarczok et al., 2022).

Heart rate variability can be described using time‐domain, frequency‐domain and Nonlinear measurements (Sassi et al., 2015; Stein and Reddy, 2005). Time‐domain quantifies the amount of variability between successive beats whereas frequency‐domain parameters estimates the distribution of power into frequency bands (Shaffer and Ginsberg, 2017). Nonlinear measurements ensure the unpredictability of a time series can be quantified (Stein and Reddy, 2005). Altered HRV is associated with myocardial infarction, (Lombardi et al., 1996) hypertension, (Schroeder et al., 2003) diabetes (Singh et al., 2000) and chronic heart failure (La Rovere et al., 2003) and is a predictor of mortality in heart failure (Tapanainen et al., 2002). HRV is reduced in people with heart failure (Jian et al., 2019). Low values of HRV have been associated with increased mortality amongst the elderly and is a more powerful predictor of mortality than conventional risk factors, such as smoking and sex in elderly individuals (Huikuri et al., 1998).

Quantification of cardiac function has traditionally been based on measures such as left ventricular ejection fraction (LVEF). The LVEF has its limitations as it is load‐dependent, subjective and affected by heart rate, it does not measure myocardial contraction and is not sensitive enough to detect subclinical changes in the myocardium (Stokke et al., 2017). To overcome these limitations, speckle‐tracking echocardiography (STE) has gained significant clinical interest in recent years (Abou et al., 2020). The STE is a grey‐scale imaging technique which identifies bright speckles that are degenerated through the interaction of ultrasound with the myocardium. Once detected, the speckles are tracked frame‐by‐frame, thus providing a direct assessment of myocardial movement (Abou et al., 2020; Stokke et al., 2017). Measurement of left ventricular global longitudinal strain, derived from STE, is a novel method proven to be the most sensitive and reproducible measurement in the evaluation of LV systolic function (Dillon et al., 1973; Karlsen et al., 2019). This imaging method discriminates between active and passive myocardial motion and allows angle‐independent quantification of myocardial deformation as the myocardium shorten and lengthens during the cardiac cycle (Abou et al., 2020; Dillon et al., 1973).

While limited number of studies report association between HRV and indices of cardiac function, it appears that only one other study investigated relationship between time‐ and frequency‐domain measures of HRV and left ventricular strain measurements in hypertensive patients (Tadic et al., 2015a). However, no studies have investigated the association between novel indices of cardiac function obtained using the STE such as right ventricular global longitudinal strain (RV‐GLS), left atrial strain, including left atrial reservoir strain (LAres), left atrial conduit strain (LAcond) and left atrial contraction strain (LAcon) along with left ventricular global longitudinal strain (LV‐GLS). Therefore, the aim of this present study was to evaluate for the first time the relationship between HRV and echocardiographic variables of cardiac function quantified by STE and those obtained using conventional volume‐based parameter including LVEF in healthy individuals.

## 2 METHODS

### 2.1 Study design

Thirty healthy adults (9 females) were recruited into this single centre, observational study Table 1. Ethical approval was obtained from Coventry University Research Ethics Committee [P109193]. All participants provided written informed consent and all study procedures were conducted in accordance with the Declaration of Helsinki. Participants with a history of chronic cardiovascular, respiratory conditions, acute or chronic neurological impairment or progressive neurological disease, use of medication which affect cardiac function, current smokers and with body mass index of >35 kg/m^2^ were excluded from the study.

**Table 1:** Physical characteristics of the study population.

Physical characteristics	Mean ± SD	Range
Age, years	33 ± 10	22−57
Male/female	21/9	
Height, cm	174.9 ± 8.5	157.5–189.5
Weight, kg	78.3 ± 14.0	49–114.4
BMI, kg/m^2^	25.5 ± 4.0	18.3–37.3
Systolic blood pressure, mmHg	124 ± 12	100–142
Diastolic blood pressure, mmHg	77 ± 8	63–98

Abbreviation: BMI, body mass index.

### 2.2 Procedure

**Figure 1:**
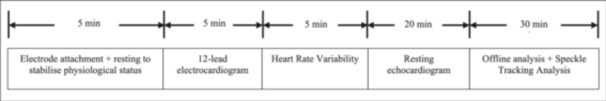
Measurement protocol for electrocardiography, heart rate variability and echocardiography.

Participants attended the Cardiovascular Research Laboratory where investigations were performed. To ensure consistency in HRV measurements, participants were asked to refrain from consuming caffeine, alcohol and vigorous exercise 24 h before the visit. Room temperature was kept at 21°C with a humidity of 34%. Blood pressure and a 12‐lead electrocardiogram was performed to confirm normal cardiac rhythm. The flow chart of the research protocol is outlined in Figure 1. Participant's anthropometric measurements were taken, and participant body mass index was calculated. Medical history was obtained to ensure participants met the inclusion and exclusion criteria of the study.

### 2.3 Heart rate variability

Measurement of HRV was in accordance with recommendations from the joint Task Force Guidelines of the European Society of Cardiology and the North American Society of Pacing and Electrophysiology (Sassi et al., 2015). Short‐term recording was used to measure HRV for 5‐min. This is the preferred method for short‐term analysis of HRV to ensure standardisation and stability of the HRV parameters (Sassi et al., 2015). Time‐domain parameters of HRV measured include: R‐R interval (representing) the average distance between two successive R waves, standard deviation of normal to normal beats (SDNN) and the square root of the mean squared differences of successive normal‐to‐normal beats (RMSSD). Frequency‐domain parameters included low‐frequency (LF) and high‐frequency (HF) power. LF power represents baroceptor activity at rest while HF power, often known as respiratory band, corresponds to the respiratory variation of heart rate. LF and HF could be directly derived from the raw R‐R interval data or transformed into normalised units by dividing with total spectral power (Heathers, 2014).

A standard ECG machine was used to record the 12‐lead ECG and HRV measurements (CardioExpress SL18A, Spacelabs Healthcare, USA) with manufacturer default sampling frequency of 100 Hz. Participants were asked to lie down in a supine position and were left for 5‐min to adjust to the environment. HRV was recorded at rest under light and noise‐ controlled conditions.

### 2.4 Echocardiography

All participants underwent a standard resting transthoracic echocardiography including speckle tracking according to the British Society of Echocardiography guidelines, performed by accredited echocardiographer using the commercially available ultrasound machine (Dobson et al., 2021) (Vivid IQ, GE Healthcare). LVEF was calculated using the Simpson's biplane method (Gutiérrez‐Chico et al., 2005). Myocardial deformation imaging was evaluated offline using the EchoPac reporting system (EchoPAC, GE Healthcare). Ventricular Global longitudinal strain was measured using apical four chamber, two chamber and three chamber views.

For STE acquisition and analysis, 2D greyscale images were analysed by the 2D Automated Function Imaging software (EchoPAC, GE Healthcare, Version 204). Optimal ECG recording was obtained during echocardiography and a minimum of three cycles were stored. High quality images were captured at a frame rate of 40 to 90 frames/s. LV‐GLS was obtained by automated STE of apical four chamber, three and two chamber views. Automated tracking alongside visual assessment was performed to ensure adequate tracking of the epicardium and endocardial borders within the region of interest (ROI). Once the ROI was generated, manual corrections were performed to fit the entire myocardial wall thickness. ROI was placed on the basal free wall, septum and the RV apex for RV‐GLS. For the STE assessment of the left atrium (LA), the endocardium was tracked automatically, and manual adjustment was made as the LA endocardium is thinner than the ventricles. Peak atrial longitudinal strain (PALS) and peak atrial contractile strain (PACS) values were obtained at the onset of the QRS complex on the ECG that is, R‐R gating.

### 2.5 Statistical analysis

All strain values are presented as absolute values for simpler interpretation. Descriptive statistics included mean ± standard deviations. All statistical analysis was carried out using IBM SPSS software, version 28. HRV and echocardiography data were visually assessed to check for normal distribution, data were then tested for normality using Kolmogorov–Smirnov or Sharipo–Wilk tests. Relationship between HRV and echocardiography indices was analysed using bivariate Pearson's coefficient of correlation (*r*). A *p*‐value < 0.05 was used to indicate statistical significance.

## 3 RESULTS

**Figure 2:**
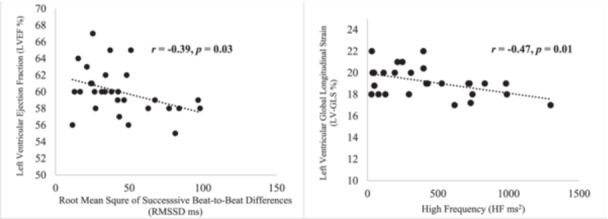
Left: Relationship between left ventricular ejection fraction (LVEF %) and root mean square of successive beat to beat difference (RMSSD ms). Right: Relationship between left ventricular global longitudinal strain and high frequency (HF ms^2^). LVEF, left ventricular ejection fraction; RMSSD, root mean square of successive differences, square root of the mean squared differences of successive normal‐to‐normal beats.

Demographic and physical characteristics of the participants are presented in Table 1. Mean values of time‐ and frequency‐domain measures of HRV are presented in Table 2. Mean values of echocardiography indices of cardiac function are presented in Table 3. Pearson's coefficient of correlation analysis revealed a significant negative relationship between HF and LV‐GLS (*r* = −0.47, *p* = 0.01) and between RMSSD and LVEF (*r* = −0.39, *p* = 0.03, Figure 2); there were no significant relationships between HRV and RV‐GLS and other measures of cardiac function (Table 4).

**Table 2:** Mean and standard deviation for heart rate variability measurements.

Heart rate variability measurements	Mean ± SD	Range
RR average	990.8 ± 175.8	739–1369
SDNN	50.9 ± 21.5	14.8–100.3
RMSSD	46.8 ± 29.4	11.5–98.1
LF	726.9 ± 608.5	59.5–2281.3
HF	415.0 ± 351.2	28.9–1298.7
LF norm	56.0 ± 19.4	8.4–98.7
HF norm	36.5 ± 18.8	12.8−78

Abbreviations: HF, high‐frequency power; HFnorm, high‐frequency power in normalised units; LF, low‐frequency power; LFnorm, low‐frequency power in normalised units; RMSSD, root mean square of successive differences, square root of the mean squared differences of successive normal‐to‐normal beats; RR average, mean time between R waves; SDNN, standard deviation of normal‐to‐normal beats.

**Table 3:** Mean and standard deviation for echocardiography measurements.

Echocardiography indices	Mean ± SD	Range
LV‐GLS	19.2 ± 1.37	17−22
LV‐EF	59.9 ± 2.78	55–67
RV‐GLS	21.7 ± 2.69	18−27
LA_res_	36.8 ± 6.99	30−41
LA_con_	26.2 ± 7.00	23–32
LA_CT_	12.3 ± 3.56	10–15

Abbreviations: LA_con_, left atrial conduit; LA_CT_, left atrial contraction; LA_res_, left atrial reservoir; LVEF, left ventricular ejection fraction; LV‐GLS, left ventricular global longitudinal strain; RV‐GLS, right ventricular global longitudinal strain.

**Table 4:** Relationship between heart rate variability and echocardiographic measurements.

	Heart rate variability													
	RR Average		SDNN		RMSSD		LF		HF		LFnorm		HFnorm	
Echocardiography	*r*	*p*	*r*	*p*	*r*	*p*	*r*	*p*	*r*	*p*	*r*	*p*	*r*	*p*
LV‐GLS	0.11	0.54	0.14	0.47	0.00	0.99	0.04	0.84	−0.47	0.01*	0.21	0.26	−0.09	0.60
LVEF	−0.14	0.46	−0.24	0.22	−0.39	0.03*	−0.12	0.53	−0.29	0.12	0.25	0.18	−0.22	0.25
RV‐GLS	−0.06	0.76	0.19	0.30	0.20	0.29	−0.00	0.97	0.18	0.35	0.04	0.82	0.03	0.87
LA_res_	−0.24	0.20	0.34	0.07	0.19	0.31	0.07	0.72	0.29	0.33	−0.18	0.33	0.28	0.14
LA_con_	−0.28	0.14	0.04	0.82	0.05	0.79	−0.11	0.55	0.18	0.35	−0.28	0.13	0.36	0.05
LA_CT_	−0.02	0.30	0.25	0.18	0.01	0.96	0.10	0.60	0.02	0.93	0.30	0.12	−0.25	0.19

Abbreviations: HF, high‐frequency power; HFnorm, high‐frequency power in normalised units; LA_res_, left atrial reservoir; LA_con_, left atrial conduit; LA_CT_, left atrial contraction; LF, low‐frequency power; LFnorm, low‐frequency power in normalised units; LVEF, left ventricular ejection fraction; LV‐GLS, left ventricular global longitudinal strain; RMSSD, root mean square of successive differences, square root of the mean squared differences of successive normal‐to‐normal beats; RR average, mean time between R waves; RV‐GLS, right ventricular global longitudinal strain; SDNN, standard deviation of normal to normal beats.

* indicates a *p*‐value of <0.05.

## 4 DISCUSSION

The present study evaluated the relationship between HRV and novel echocardiographic indices of cardiac function. Our findings suggest that HRV (RMSSD and HF) is associated with measures of left ventricular function, such as LV‐LGS and LVEF. This provides better understanding of the interaction between autonomic and contractile function of the heart. In the present study, HF power and RMSSD, both indicators of parasympathetic activity, were inversely related to LV‐GLS and LVEF respectively. This confirms previous research reporting the inhibitory effects of parasympathetic activity on cardiac contractility (Buchheit et al., 2010; Vaseghi and Shivkumar, 2008). The mean values for HRV indices reported in this study are similar to those reported in a study involving healthy adults participants (Nunan et al., 2010).

A limited number of studies have assessed the relationship between HRV and cardiac functional and morphological properties, majority of the studies conducted in clinical populations (Hayano et al., 2021; Kleiger et al., 1987; Nolan et al., 1998; Stein and Reddy, 2005). For example, Shehab et al., 2009 showed that SDNN was not related to left ventricular systolic dysfunction in patients who presented with noncardiac vascular episodes (Shehab et al., 2009). In contrast, a negative relationship was seen in a large cohort study of 7983 participants free of heart failure and atrial fibrillation, where every unit increase in RMSSD was associated with lower LVEF (Arshi et al., 2022). Further research is warranted to assess if lower RMSSD values are also associated with measures of left ventricular function in younger healthy individuals with no risk factors, such as the cohort of this study. The interpretation and generalisation of HRV in diseased populations and individuals with normal cardiac function may not be straightforward, (De Jong and Randall, 2005) as different populations will have different reference ranges. Only one other study utilised STE to assess the relationship between LV function and HRV in untreated hypertensives and healthy controls, which reported a relationship between both across the whole study population (Tadic et al., 2015a). However, although this study evaluated the relationship between HRV and cardiac mechanical function using STE, the novelty of the present study explored the relationship between not only left ventricular function but also right ventricular and left atrial function.

Previous studies reported that HRV values (i.e. RMSSD) had an association with LVEF, those with heart failure and ischemic heart disease had lower HRV measurements compared to age and gender matched healthy controls (Alkhodari et al., 2021; Tian et al., 2022). HRV has been reported to be lower in patients with reduced LVEF with underlying pathologies including MI (Hayano et al., 2021) and chronic heart failure (Musialik‐Łydka et al., 2003). Increased sympathetic function is associated with reduced cardiac function and can trigger arrhythmias, (Carnagarin et al., 2019) though, parasympathetic nervous system (PNS) has a protective effect on the cardiovascular system (Wharton et al., 1992). Under resting physiological conditions, PNS activity is predominant and regulates heart rate and blood pressure (Wharton et al., 1992). Whilst the present study did not find a relationship between HRV, right ventricular function, and left atrial function, previous studies reported relationships between HRV and right ventricular remodelling in patients with untreated hypertension, (Tadic et al., 2015b) and left atrial reservoir function in asymptomatic diabetic patients (Tadic et al., 2017). Our study population were healthy, and this is a likely reason for our findings. Under pathological conditions, mechanical changes precedes electrical changes (Poli et al., 2019). Therefore, alterations in HRV occur after changes in cardiac muscle function. Thus, it is not surprising that in healthy participants, no significant association was observed between HRV and left atrium and right ventricle function. Medenwald et al., 2017 concluded that no association was seen between left atrial systolic dimension, LVEF and HRV measurements (Medenwald et al., 2017). It could be argued that the method of calculating EF in this group was through Teichholz and not through Simpson's biplane method. Although, only HF and RMSSD, which are associated with the parasympathetic component of the autonomic nervous system, had a relationship with the left ventricle (Ben Mrad et al., 2021).

Although the use of HRV is widely recognised in various disease groups, it has not been used widely in clinical practice. Whether autonomic derangement occurs before cardiac dysfunction or is a consequence of reduced LV function is unknown. However, these two aspects are related, and reduced HRV may be a novel sign of reduced cardiac function (Shah et al., 2013). This notion is supported with findings from the present study confirming the relationship between HF power and LV‐GLS and RMSSD and LVEF. HRV has been reported to be a strong predictor of mortality, (Hayano et al., 2021) particularly in those with reduced LVEF when mortality was five‐times higher (Kleiger et al., 1987). Furthermore, reduced HRV predicted sudden arrhythmogenic events in post‐MI patients (Liu et al., 2020). As an independent measure, LVEF alone cannot predict mortality, (Chattipakorn et al., 2007) therefore, LV‐GLS can be utilised as a more sensitive marker for LV dysfunction (Smiseth et al., 2016).

The findings from the present study suggest there was a significant negative relationship between HF power, RMSSD and measures of LV systolic function in healthy individuals. The relationship between HRV as a measure of autonomic nervous system and left ventricular systolic function appears to extend beyond the diseased populations to the healthy state as seen in this study. This relationship is confirmed in this study using STE and which did not include LA and RV function. As normal LV function is related to normal reference ranges of HRV measurements, it could be suggested that HRV measurements may be used as a predictor of systolic function of the heart and therefore contribute to improvements in risk stratification, prognosis and mortality (Fang et al., 2020; Vuoti et al., 2021) and reduced the risk of sudden cardiac death and all cause mortality (Sessa et al., 2018).

A major limitation of the present study was the use of healthy individuals; therefore, our findings should not be generalised to clinical populations. This study only assessed relationships between variables therefore, cause and effect cannot be established. However, the present study improves the understanding of the human physiology and link between HRV and measures of cardiac function, its major findings should not be generalized due to modest sample size and focus on healthy population. Nonetheless, it is valuable to investigate such relationships in healthy populations before investigating clinical populations as pathology could make results difficult to interpret.

## 5 CONCLUSION

Parasympathetic activity of the autonomic nervous system, represented by high frequency power and RMSSD of the HRV is associated with left ventricular systolic function in healthy individuals. Thus, it is reasonable to suggest that HRV is a practical noninvasive measure that can be used as an indicator of left ventricular function. Further research is warranted to evaluate the utility of HRV for monitoring cardiac function, and to identify thresholds which will facilitate risk stratification and prognosis in general and clinical populations.

## AUTHOR CONTRIBUTIONS

**Mushidur Rahman**: Formal analysis; investigation; writing—original draft; writing—editing. **Sophie L. Russell**: Investigation; writing—review and editing. **Nduka C. Okwose**: Formal analysis; investigation; writing—review and editing. **Charles J. Steward**: Investigation; writing—review and editing. **Helen Maddock**: Writing—review and editing. **Pritwish Banerjee**: Writing—review and editing. **Djordje G. Jakovljevic**: Conceptualisation; funding acquisition; investigation; methodology; project administration; writing—review and editing.

## CONFLICT OF INTEREST STATEMENT

The authors declare no conflict of interest.

## Data Availability

All data will be available to all authors upon reasonable request.
